# Effects of substrates and suppliers of ingredients on microbial community and metabolites of traditional non-salt Suancai

**DOI:** 10.20517/mrr.2023.76

**Published:** 2024-03-13

**Authors:** Haimei Lai, Lang Yan, Yali Wang, Yuan Mei, Yuli Huang, Xueqing Zeng, Lihong Ge, Jichun Zhao, Yongqing Zhu, Qiaolian Huang, Menglu Yang, Nan Zhao

**Affiliations:** ^1^Institute of Agro-products Processing Science and Technology, Sichuan Academy of Agricultural Sciences, Chengdu 610066, Sichuan, China.; ^2^Panxi Crops Research and Utilization Key Laboratory of Sichuan Province, Xichang College, Xichang 615000, Sichuan, China.; ^3^College of Life Science, Sichuan Normal University, Chengdu 610066, Sichuan, China.; ^4^College of Food Science, Southwest University, Chongqing 400715, China.

**Keywords:** Non-salt Suancai, substrate/supplier of ingredients, metabolites, microbial community

## Abstract

**Aim:** Non-salt Suancai is an acidic fermented vegetable consumed by the Chinese Yi ethnic group. Traditionally, it is produced by fermentation without salt in a cold environment. The present study aimed to investigate the metabolite and microbial characteristics, and the effects of substrates/suppliers ingredients on non-salt Suancai.

**Methods:** A simulated fermentation system of non-salt Suancai was constructed by using different substrates/suppliers’ ingredients. The coherence and differential detection of the metabolite and microbial characteristics were done through non-target metabolomic and metagenomic analysis.

**Results:** Lactic acid was the predominant organic acid across all samples. The enumeration of the Lactic acid bacteria showed no discernible differences between study groups, but that of yeast was highest in the mustard leaf stem (*Brassica juncea* var. *latipa*). The three major biological metabolic pathways were metabolism, environmental information, and genetic information processing based on the KEGG database. The metabolite diversity varied with the substrate/supplier of ingredients based on the PLS-DA plot. *Lactiplantibacillus*, *Leuconostoc*, and *Lactococcus* were prevalent in all samples but differentially. The microbial diversity and richness varied significantly, with 36~291 species being identified. Among the various substrates collected from the same supplier, 29, 59, and 29 differential species were identified based on LEfSe [linear discriminant analysis (LDA) > 2, *P* < 0.05]. *Leuconostoc citreum*, *Leuconostoc mesenteroides*, *Leuconostoc pseudomesenteroides*, *Lactiplantibacillus plantarum*, and *Leuconostoc lactis* were likely to be used as the species to discriminate samples collected from different suppliers.

**Conclusions:** This research contributed to the exploration of microbial and metabolite characteristics behind the ingredient restriction of non-salt Suancai using traditional technology.

## INTRODUCTION

Non-salt Suancai is a traditional fermented vegetable (*Brassica* L.), which is widely consumed in the southwest of Sichuan Province (the Yi ethnic group), China for its acidic taste. In contrast to most Suancai consumed in other regions, non-salt Suancai can be rapidly fermented without salt and naturally air-dried in winter (8 °C ~ 16 °C). The key method for fermentation of Suancai is the boiling of raw materials followed by the addition of fermentation starter (preserved dried Suancai) [Supplementary Figure 1]. Thermally treated root vegetables enhance the release of polysaccharides, especially that of β-glucans and pectic components, and increase the viscosity^[1]^. Starter culture is a frequently used technique to control and improve the quality of fermented products^[2]^. Thus, the typical way of using preserved dried Suancai as a fermentation starter is an effective method to supply complex and naturally selected microbiota. These microbiota can accelerate the fermentation process and regulate the quality of the product^[3]^. This technique of producing non-salt Suancai gives it a distinctive feature with a strong sour fragrance and a sliding-back sweet taste. However, the quality characteristics of non-salt Suancai with the special processing technology are still to be explored.

Fermentation represents complex high-order microbial interactions. Notably, the microbial terroir of grapes has been linked to its origin, which also determines the regional variation of wine^[4]^. This concept also aligns with the research on Daqu, a traditional Chinese liquor fermentation starter, which contains abundant microbiota that contribute to its metabolite profile^[5]^. A significant amount of evidence suggests that terroir influences metabolites, microbes, and sensory characteristics in kimchi, a Korean vegetable product^[6]^. Therefore, the microbial terroir of preserved dried Suancai varies with different suppliers due to natural fermentation. The investigation of the microbial characteristics of non-salt Suancai produced by different suppliers is crucial to understanding the mechanism behind this fermentation method. The variety of active ingredients is also a critical factor that influences the quality of the fermented product. The inherent attributes^[7]^ and the recognizable strains^[8]^ differ with the substrate of ingredients, and the difference in substrates further alters the quality of fermented products. For example, metagenomic analysis reveals that differences in amino acid and carbohydrate metabolism pathways between sorghum substrates alter the volatility of Chinese strong-flavored Baijiu^[9]^. Similarly, within fermented vegetables, the substrate of garlic influences the microbial ecology and metabolite profile of kimchi^[10]^, and the selection of chili pepper had a significant effect on the physiochemical property and the flavor of Paojiao, a fermented Chinese chili pepper^[11]^. However, there is a lack of knowledge regarding the effects of different substrates/supplier patterns on non-salt Suancai.

Metabolomics involves comprehensive identification and quantification of small molecules (≤ 1,500 Da). When typically coupled with nuclear magnetic resonance (NMR) or mass spectrometry (MS) with upstream separation methods, it can improve the understanding of the quality attribute of foods at the molecular level^[12]^. For example, metabolomic analysis revealed both targeted and untargeted metabolites of fermented soybean foods, which suggested their role in flavor development and therapeutic properties of them^[13]^. The shelf life of green tea beverage could be predicted by identifying markers determined by metabolomic analysis^[14]^. In addition, the variation of metabolites during cold storage of Torreya yunnanensis seeds was investigated through metabolomic analysis, which provided insights into conservation efforts and habitat restoration^[15]^.

To reveal the microbial and metabolite characteristics of non-salt Suancai, the simulated fermentation system was constructed. Furthermore, the effects of different substrates and suppliers of ingredients on microbial counts, physicochemical properties, organic acids, metabolites, and microbial community were also evaluated. This is the first study investigating the non-salt Suancai and its microbial and metabolite characteristics. Therefore, the present study contributes to the scientific inheritance of traditional technology and provides the basis for quality control.

## MATERIALS AND METHODS

### Sample preparation

Fresh vegetables were purchased from a local supermarket and used to produce non-salt Suancai. The preserved dried Suancai was purchased from different local suppliers in Xichang City, Sichuan Province, China (101.97° E, 27.93° N), which was used as a starter for fermentation. There were five Manjing leaves (MF) (*Brassica rapa* L.), one Manjing rhizome (MR), and one mustard leaf stem (MLS) (*Brassica juncea* var. *latipa*). Fresh vegetables were boiled for over 5 min and then transferred to a bottle containing different preserved dried Suancai. The fermentation was done at room temperature (10 ± 2 °C). After 15 days of fermentation, the fermented vegetables and liquids were evaluated. To differentiate the sample information, samples collected from the same suppliers were numbered MF-A, MR-A, and MLS-A according to their substrate ingredients. The other four purchase channels (suppliers) of samples were labeled as MF-B, MF-C, MF-D, and MF-E, respectively, which were all made by Manjing leaf (*Brassica rapa* L.).

### Microbial counts and physicochemical properties assessment

The fermented liquid of each sample was serially diluted in normal saline (NaCl, 0.85%, w/v), and the diluent was plated on de Man, Rogosa and Sharpe (MRS; Oxoid Ltd, Hampshire, UK) and Rose Bengal agar (RBA; Qingdao Hope Bio-Technology Co., Ltd, China) to enumerate lactic acid bacteria and yeast, respectively^[16]^. MRS plates were incubated at 37 °C for 48 h, and Rose Bengal agar plates were incubated at 30 °C for 48 h. Each microbial count was recorded as log colony-forming units per gram of sample (log CFU/mL). The total acid (TA) and reducing sugar (RS) contents of the fermented vegetable samples were measured. Briefly, 2 g of each sample was subjected to ultrasonic (40 kHz, room temperature) extraction with ultrapure water, and the mixture was filtrated (qualitative grade filter circles) to obtain supernatant for further analysis. TA and RS were determined based on Chinese national standards (GB 12456-2021 and GB 5009.7-2016), which refer to the acid-base titration method and the 3,5-dinitrosalicylic acid (DNS) method, respectively. Total sugar (TS) was analyzed by a modified procedure of a previous publication^[17]^: a 5 g sample was mixed with 100 mL HCl (2%, v/v) and treated at 121 °C for 30 min. Then, the pH of the extraction solution was adjusted to neutrality with NaOH (20%, w/v) after cooling down. The solution was then diluted to 250 mL and filtered using filter paper.

### Analysis of organic acids

High-performance liquid chromatography (Agilent G1260 HPLC, Agilent Technologies Co. Ltd., USA) was used to analyze organic acids (OAs) based on a previous publication^[18]^ but with some optimizations. For fermented mustard, a 3 g sample was mixed and homogenized with 0.004 M H_2_SO_4_ at a ratio of 2:7 (w/v), followed by extraction for 30 min. Prior to analysis, the treated sample was centrifuged at 12,000 r/min for 10 min, and 1 mL of the supernatant was filtered through a 0.22 μm filter membrane. OAs separation was done on an Aminex HPX-87H column (300 mm × 7.8 mm, Bio-Rad) at a column temperature of 35 °C using H_2_SO_4_ (0.004 M) as the mobile phase at a wavelength of 215 nm. The injection volume was 20 μL and the flow rate was adjusted to 0.6 mL/min.

### Non-target metabolomics analysis

The fermented mustards (100 ± 5 mg) were mixed with 400 μL extraction solution (80% methanol-water solution, v/v) containing 0.02 mg/mL L-2-chlorophenylalanine (internal standard). Samples were separately ground using Wonbio-96c frozen tissue grinder (Shanghai Wanbo Biotechnology Co., LTD.) for 6 min (-10 °C, 50 Hz), and then extracted by low-temperature ultrasound for 30 min (5 °C, 40 kHz). Subsequently, the sample was centrifuged for 15 min (4 °C, 13,000 *g*) after being left at -20 °C for 30 min, and the supernatant was transferred to the injection vial for ultra-high performance liquid chromatography-tandem mass spectrometry (UPLC-MS/MS) analysis. In addition, the mixture of each supernatant of the sample (20 μL) was used as a quality control (QC).

As described in a previous study^[19]^, UPLC-MS/MS analyzers were carried out with a Thermo UHPLC-Q Exactive HF-X system equipped with an ACQUITY UPLC BEH C18 column (100 mm × 2.1 mm, i.d., 1.7 μm; Waters, Milford, USA). Non-targeted metabolomics was conducted by Majorbio Bio-Pharm Technology Co. Ltd. (Shanghai, China). In brief, the mobile phase A was 2% acetonitrile-water (containing 0.1% formic acid) and B was acetonitrile (containing 0.1% formic acid). The injection volume was 3 μL. The flow rate was 0.40 mL/min and the column temperature was 40 °C. The mobile phase elution gradient is presented in Supplementary Table 1. Mass spectrometric data were collected using an electrospray ionization (ESI) source operating in positive and negative modes. Optimal conditions were set as follows: heater temperature at 450 °C, capillary temperature at 320 °C, sheath gas flow rate at 50 arb, Aux gas flow rate at 13 arb, and ion-spray voltage floating (ISVF) at -3,000 V in negative mode and 3,500 V in positive mode, respectively. The normalized collision energy was 20-40-60 V rolling for MS/MS. Full MS resolution was 70,000 and MS/MS resolution was 17,500. Data acquisition was performed in Data Dependent Acquisition (DDA) mode. Detection was performed over a mass range of 70-1,050 m/z.

Raw LC/MS data were pre-processed using Progenesis QI software (Waters Corporation, Milford, USA). The data matrix was pre-processed to retain at least 80% of the metabolic signatures detected in each sample. Raw and annotated data for metabolomics are reported in Supplementary Table 2. Then, the specific sample was estimated as the minimum metabolite value, and each metabolic signature was normalized to the sum. Metabolites were then identified by searching databases [HMDB (http://www.hmdb.ca/), Metlin (https://metlin.scripps.edu/), and Majorbio (https://cloud.majorbio.com)]. Meanwhile, the variables of the QC samples with relative standard deviation (RSD) > 30% were excluded and converted to log10 values to obtain final data matrix for subsequent analysis. Differential metabolites between the two groups were mapped to their biochemical pathways by metabolic enrichment and pathway analysis based on the KEGG database (http://www.genome.jp/kegg/). Statistical analysis was performed using Python package “scipy.stats” (https://docs.scipy.org/doc/scipy/) to obtain the most relevant biological pathways for experimental groups.

### Metagenomic analysis

Metagenomic sequencing was performed based on previously published methods^[20]^. The fermented liquid was centrifuged for 10 min (4 °C, 10,000 *g*) to enrich microorganisms. The total microbial genomic DNA of the samples was extracted using E.Z.N.A® Soil DNA Kit (Omega Biotek, USA), and then the quantity and quality of extracted DNAs were determined. Total DNA was fragmented using Covaris M220 and 400 bp fragments were screened. The paired-end library was constructed using NEXTFLEX^TM^ Rapid DNA-Seq (Bioo Scientific, Austin, TX, USA). Illumina NovaSeq 6000 (Illumina Inc., San Diego, CA, USA) from Majorbio Bio-Pharm Technology Co., Ltd. (Shanghai, China) was used for paired-end sequencing. Experiments were performed in triplicate, with samples 1, 2, and 3 representing the biological replicates.

Fastp (https://github.com/OpenGene/fastp, version 0.20.0) was used to cut the adapter of the raw reads and remove low-quality reads (read length less than 50 bp, average base mass value less than 20, or reads contain N base). Host DNA sequences were removed using the BWA software (http://bio-bwa.sourceforge.net, version 0.7.9a). The high-quality reads were then assembled into contigs using MEFAHIT (https://github.com/voutcn/megahit, version 1.1.2), and then the screened contigs (≥ 300 bp) were further used for ORF prediction [ORF finder (https://www.ncbi.nlm.nih.gov/orffinder/)]. SOAPaligner software (https://help.rc.ufl.edu/doc/SOAPaligner) was used to calculate gene abundance information. Raw and annotated data for metagenomics are shown in Supplementary Table 3. The annotation of the amino acid sequence of the non-redundant gene catalog based on the NCBI NR database was obtained by Diamond (https://v2.pseudomonas.com/blast/setpdiamond), as well as the calculation of species abundance. In addition, the corresponding function of genes was revealed by comparing it with the KEGG database (https://www.kegg.jp/) with an e-value cut-off of 1e^-5^, and the abundance of function was calculated based on KO, Pathway, EC, and Module.

### Statistical analyses

Differential metabolites were considered by VIP > 1 (the variable importance in projection was obtained based on PLS-DA), *P*-value < 0.05 (the statistical significance was calculated based on *T*-test), and FC ≥ 2 or FC ≤ 0.5 (fold change of metabolites). PCA and PLS-DA were performed with SIMCA-P (Umetrics, Sweden). Volcano plots were used to filter metabolites of interest based on related parameters. Results are expressed as mean ± standard deviation (SD). Data were analyzed using one-way analysis of variance (ANOVA) and Duncan’s test in the SPSS 19.0 software (SPSS Inc., Chicago, IL., USA) at a significance level of *P* < 0.05. For the microbial community, similarities analysis (ANOSIM), barplot, and heatmap were produced in the Vegan package of R (version 3.3.1). Venn plot and LEfSe (linear discriminant analysis effect size) were used to recognize the common and/or unique species.

## RESULTS AND DISCUSSION

### Differences in microbial counts and physicochemical properties

The microbial counts could reflect the activity of fermentation, in which lactic acid bacteria and yeast counts are shown in Table 1. Both of these organisms were the main fermentative candidates in non-salt Suancai. The count of LAB in MLS-A was slightly lower than that in MF-A and MR-A. It was probably associated with the content of isothiocyanates in different varieties of *Brassica* ingredients belonging to the Cruciferae plants, which possess antibacterial properties^[21]^. The LAB counts in samples were consistent with that of Laotan Suancai, another well-known Chinese traditional fermented vegetable^[22]^, which indicated that the system of non-salt Suancai was also a typical LAB fermentation. However, the tendency of the yeast counts was opposite. The yeast count in MLS-A was relatively high at 5.04 log CFU/mL, while that was 2.34 and 2.67 log CFU/mL in MF-A and MR-A, respectively. Yeast can promote flavor in Paocai^[23]^, while the massive amount of film yeast is the inducer of pellicle formation^[24]^. In addition, yeast counts of samples made by Manjing (*Brassica rapa* L.) were roughly the same and relatively low.

**Table 1:** Differences in microbial counts and detection indexes

**Indicators**	**MLS-A**	**MR-A**	**MF-A**	**MF-B**	**MF-C**	**MF-D**	**MF-E**
LAB counts / Log CFU/mL	8.23 ± 0.05^a^	8.54 ± 0.02^b^	8.54 ± 0.07^b^^,^^c’^	7.78 ± 0.09^a’^	7.90 ± 0.08^b’^	8.56 ± 0.03^d’^	8.56 ± 0.08^d’^
Yeast counts/Log CFU/mL	5.04 ± 0.01^c^	2.67 ± 0.03^b^	2.34 ± 0.08^a^^,^^d’^	2.48 ± 0.10^e’^	1.30 ± 0.05^a’^	2.04 ± 0.08^c’^	1.60 ± 0.07^b’^
TA / g/100g	0.60 ± 0.03^b^	0.61 ± 0.01^c^	0.58 ± 0.02^a^^,^^e’^	0.29 ± 0.01^a’^	0.45 ± 0.01^b’^	0.51 ± 0.01^d’^	0.50 ± 0.01^c’^
TS g/100g	0.53 ± 0.07^a^	0.73 ± 0.07^c^	0.55 ± 0.08^b^^,^^c’^	0.37 ± 0.05^a’^	0.56 ± 0.22^d’^	0.56 ± 0.22^d’^	0.38 ± 0.09^b’^
Lactic acid / g/kg	10.11 ± 0.37^c^	9.13 ± 0.14^a^	9.43 ± 0.17^b^^,^^c’^	5.43 ± 0.03^a’^	7.41 ± 0.15^b’^	10.04 ± 0.03^e’^	9.67 ± 0.25^d’^
Acetic acid / g/kg	0.39 ± 0.01^a^	0.95 ± 0.04^c^	0.70 ± 0.03^b^^,^^c’^	0.81 ± 0.02^e’^	0.63 ± 0.02^b’^	0.42 ± 0.02^a’^	0.75 ± 0.05^d’^

a, b, and c: Means ± standard deviation (*n* = 3) with different letters within a row are significantly different (*P* < 0.05) between the different substrates of ingredients (MLS, MR, and MF). Make a comparison between the different suppliers (MF-A ~ E) and marked as: a’, b’, c’, d’, and e’ (*P* < 0.05). MLS: Mustard leaf stem; MR: Manjing rhizome; MF: Manjing leaves; LAB: lactic acid bacteria, TA: total acids, TS: total sugars.

RS was exhausted in all samples after 15 days of fermentation, indicating that the microbes grew well and reached the end of fermentation. In general, microorganisms can utilize RS and produce acids through carbon cycle^[25]^. As shown in Table 1, the substrate of ingredients had no effects on TA content, while it varied with the suppliers. MF-B showed the lowest TA content, consistent with its LAB count. It is worth mentioning that the phenomenon of sugar filaments was displayed in MF-A, MLS-A, MR-A, MF-C, and MF-D. TS index was used to characterize filament phenomenon. Results showed that the content of TS was relatively high in those samples with sugar filament, which made us speculate that the filament appearance is mainly associated with a specific microbiota no matter what substrates/suppliers of ingredients are.

### Differences in organic acids profile

Citric acid, succinic acid, and malic acid were not detected in any of the samples, which could have been metabolized by microbes to get energy and produce flavor^[18]^. Lactic acid and acetic acid were the main OAs in non-salt Suancai, and their contents are listed in Table 1. The content of lactic acid was roughly the same among different substrate samples, while the acetic acid content in MLS-A was lower than that in MF-A and MR-A. Among the same substrate samples, a relatively low content of lactic acid was detected in MF-B and MF-C, which was consistent with their LAB counts. Interestingly, MF-D showed the highest content of lactic acid and the lowest content of acetic acid, which implies that a suitable flux of homolactic and heterolactic fermentation occurred in the Suancai system and was related to LAB strains^[26]^.

### Non-target metabolomics analysis

#### Differences in metabolite profiles

A total of 510 metabolites were identified in all samples for the positive mode and 317 metabolites in the negative mode. PCA was performed on the peaks detected in the experimental and QC samples, and the QC samples were clustered together on the PCA diagrams [Supplementary Figure 2A] to ensure the quality of the measured data. There were 809 shared metabolites identified in all samples, and there were no exclusive metabolites [Supplementary Figure 2B]. In the phytochemical classification of metabolites, the total proportion of primary (287) and secondary (315) metabolites was 74.13%. Lipids, carbohydrates, and amino acids and derivatives were the three most abundant primary metabolites [Supplementary Figure 3A], accounting for 94.43% of the total primary metabolites. The major secondary metabolites were flavonoids, phenolic acids, terpenoids, organic acids, indoles, and coumarins and their derivatives, all of which accounted for 89.53% of the total secondary metabolites [Supplementary Figure 3B].

The metabolites were further enriched into corresponding metabolic pathways based on the KEGG database and annotation information. They mainly involved three biological metabolic pathways - metabolism, environmental information, and genetic information processing [Supplementary Figure 4A]. Biosynthesis of various plant secondary metabolites, tyrosine metabolism, and ABC transporters were more active and involved more metabolites [Supplementary Figure 4B]. In addition, the metabolites were classified using the HMDS database. The most abundant metabolites were lipids and lipid-like molecules (208), followed by organoheterocyclic compounds (132) and fatty acyls (124) [Supplementary Figure 4C]. It is worth noting that the nucleotide metabolism was active, which was probably associated with intracellular nucleotide-related compounds^[27]^. In addition, nucleotide metabolism was also susceptible to inactivation of microbes, peptidoglycan synthesis, and amino acid metabolism^[28]^. Therefore, metabolism was simultaneously related to growth and occurred in the inactivation process of microbes.

#### Differences in metabolites based on multiple samples comparison

There were 21 (MF-B), 19 (MF-C), 25 (MF-D), 24 (MF-E), 23 (MLS-A), 25 (MF-A), and 22 (MR-A) metabolites whose relative content was higher than 1% in each sample. Queuine, cyclopentanol, pc (16:0/0:0), palmitic acid, isorhamnetin, fisetin, and kaempferol 3-o-sophoroside were universally predominant metabolites (the relative content > 3%) in non-salt Suancai. Among them, queuine was highlighted in MF-B (5.24%) and MF-C (13.30%), which was derived from a de novo synthesized metabolite in bacteria^[29]^. Moreover, the relative content of cyclopentanol, isorhamnetin, pc (16:0/0:0), palmitic acid, and pyroglutamic acid was higher in MF-D, MF-E, MLS-A, MF-A, and MR-A, respectively, among which cyclopentanol endows pleasant flavors and isorhamnetin possess antioxidant properties^[30]^. Palmitic acid and pyroglutamic acid were involved in fatty acid metabolism and glutathione metabolism, respectively.

**Figure 1:**
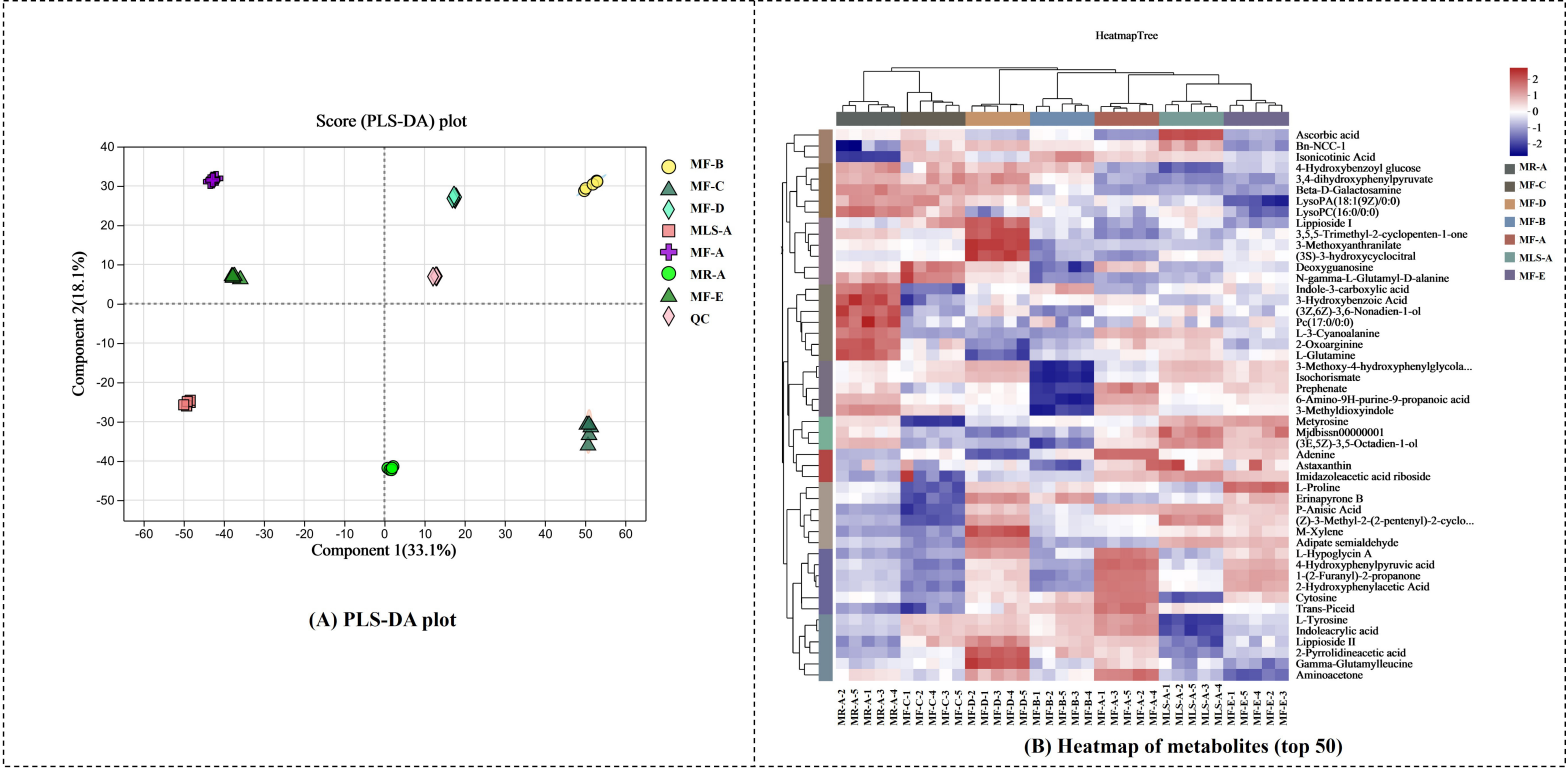
(A) Overview pattern of the metabolite differences based on PLS-DA score plots; (B) heatmap of metabolites. All of the metabolites were calculated in the PLS-DA plot, and the heatmap shows metabolites whose relative content was in the top fifty.

The overall patterns of metabolite differences in non-salt Suancai fermented by different substrates/suppliers of ingredients are shown in PLS-DA score plots. As shown in Figure 1A, there was an obvious difference in metabolites between samples. The response permutation test confirmed the absence of overfitting and misinformation in the data. It can be concluded that the metabolic preference and metabolic diversity varied remarkably with the substrates/suppliers. Heatmap and cluster analysis illustrate the composition of top 50 metabolites [Figure 1B]. The parallel assays of each sample were clustered together, indicating good assay performance. Compared to different substrates, MR-A was clustered into a single cluster because of the high abundance of indole-3-carboxylic acid, 3-hydroxybenzoic acid, (3Z,6Z)-3,6-nonadien-1-ol, and Pc (17:0/0:0). Among them, indole-3-carboxylic acid was involved in the biogenesis of ascorbigen in *Brassica oleracea* L.^[31]^, the bulb of which was similar to Manjing rhizome. 3-Hydroxybenzoic acid is an antioxidant phytochemical^[32]^. Interestingly, (3Z,6Z)-3,6-nonadien-1-ol endows a strong and waxy flavor to fresh vegetables with a creamy taste, which is probably the reason for an overall flavor difference of MR. In addition, MF-C was classified into a single cluster as compared to the other suppliers produced by the same MF substrate. Deoxyguanosine and N-gamma-L-glutamyl-D-alanine showed a high abundance, whereas L-proline, erinapyrone B, p-anisic acid, and (Z)-3-methyl-2-(2-pentenyl)-2-cyclopenten-1-one were relatively low in MF-C. Therefore, it was worth noting that the microbial activity and flavor profile were different because of discrepancies in suppliers.

**Figure 2:**
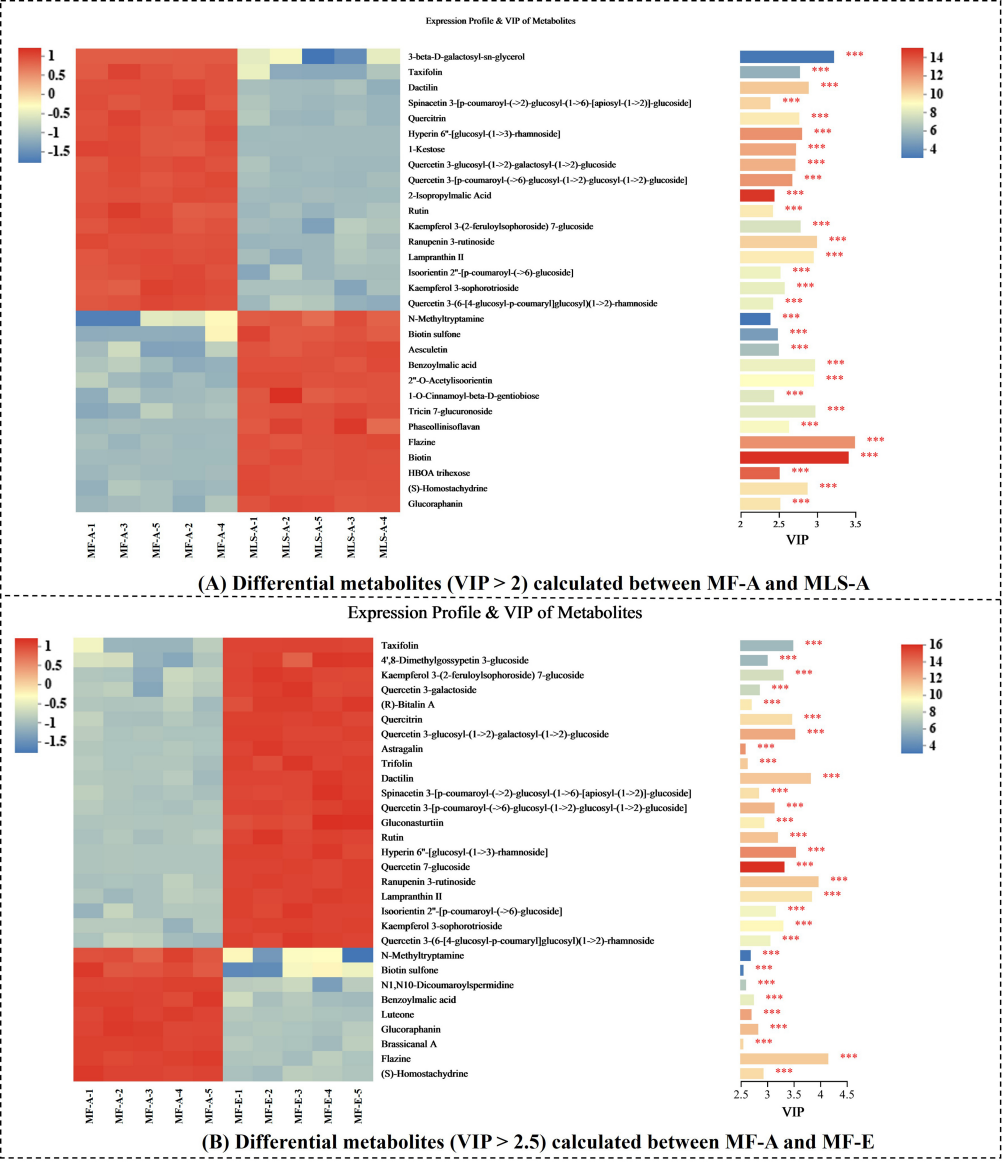
Differential metabolites calculated by OPLS-DA between (A) MF-A and MLS-A and (B) MF-A and MF-E. The selection criteria of the compounds were those whose VIP value was greater than 2 (A) or 2.5 (B). The principle of sample selection was based on the cluster results in Figure 1B, which were classified into different clusters. Asterisk refers to statistical significance, ^***^P < 0.001. MF: Manjing leaves; MLS: mustard leaf stem; VIP: variable important in projection.

According to the cluster analysis, MF-A and MLS-A were classified into an identical group, both collected from the same supplier with discrepancies in substrates. Therefore, to determine the markers for differentiating the differences between the metabolites of the two samples, the Euclidean algorithm based on OPLS-DA was applied to mark the differential metabolites (*P* < 0.05) whose abundance matched top thirty. As shown in Figure 2A, the differential metabolites were determined by calculating VIP (variable important in projection) value. Taxifolin, dactilin, quercitrin, lampranthin II, and some other glucoside/rhamnoside/rutinoside compounds were highlighted in MLS-A, which were annotated to the flavonoid glycoside subclass. The native *Lactiplantibacillus plantarum* fermentation could enrich its antioxidant activity through the accumulation of flavonoids^[33]^. The regulation of dietary flavonoid intake can improve health status by reconstructing gut microbiota^[34]^. The majority of gut microbiota are associated with flavonoid transformation. For example, *Lactococcus* is involved in the C-deglycosylation of flavonoids^[35]^. Most flavonoids contribute to the color change of yellow food^[36,37]^. Thus, it was quite reasonable to presume that the flavonoid was related to the golden yellow Suancai color. In addition, the differential metabolites in MF-A compared to MLS-A were involved in most pathways, among which aesculetin was found in the class of coumarins and derivatives, which exhibit anti-inflammatory activity^[38]^. As shown in Figure 2B, the differential metabolites with VIP > 1 were used to reveal the discrepancies between the different suppliers (MF-A and MF-E). Luteone shows highest antifungal activity against food spoilers and retains its activity at low acidic pH^[39]^. Thus, more luteone in MF-A might inhibit unwanted fungal growth and help in normal fermentation process of Suancai. Taken as a whole, the metabolites of non-salt Suancai varied with substrates of ingredients and their suppliers.

### Metagenomic analysis

#### Microbial composition

A total of 40,780,878 to 51,208,942 raw reads were generated from metagenomic sequencing analysis. After quality processing and elimination of sample host gene, the effective percentage of clean reads was 99.19% to 99.46%. Sequences were assembled using Megahit, resulting in contigs that ranged from 13,850 to 82,205. All contigs were then subjected to ORF prediction and 25,981 to 110,566 ORFs were obtained. Alpha diversity was an effective index to characterize within-habitat diversity, which includes Chao1 and Simpson index. As shown in Supplementary Table 4, the microbial richness (Chao 1) was highlighted in MF-A, while the highest microbial diversity (Simpson) was in MR-A when compared between different substrates of ingredients. In addition, among different suppliers, MF-B and MF-C showed highest richness and diversity, respectively. *Lactobacillaceae* was the predominant family in all samples (58.78%~96.17%). Through species annotation [Supplementary Figure 5], 566 species were co-detected in all samples, accounting for 66.04%~94.08%, which indicated that the basic species composition was roughly the same. However, the 36~291 differential species related to the discrepancies of samples.

**Figure 3:**
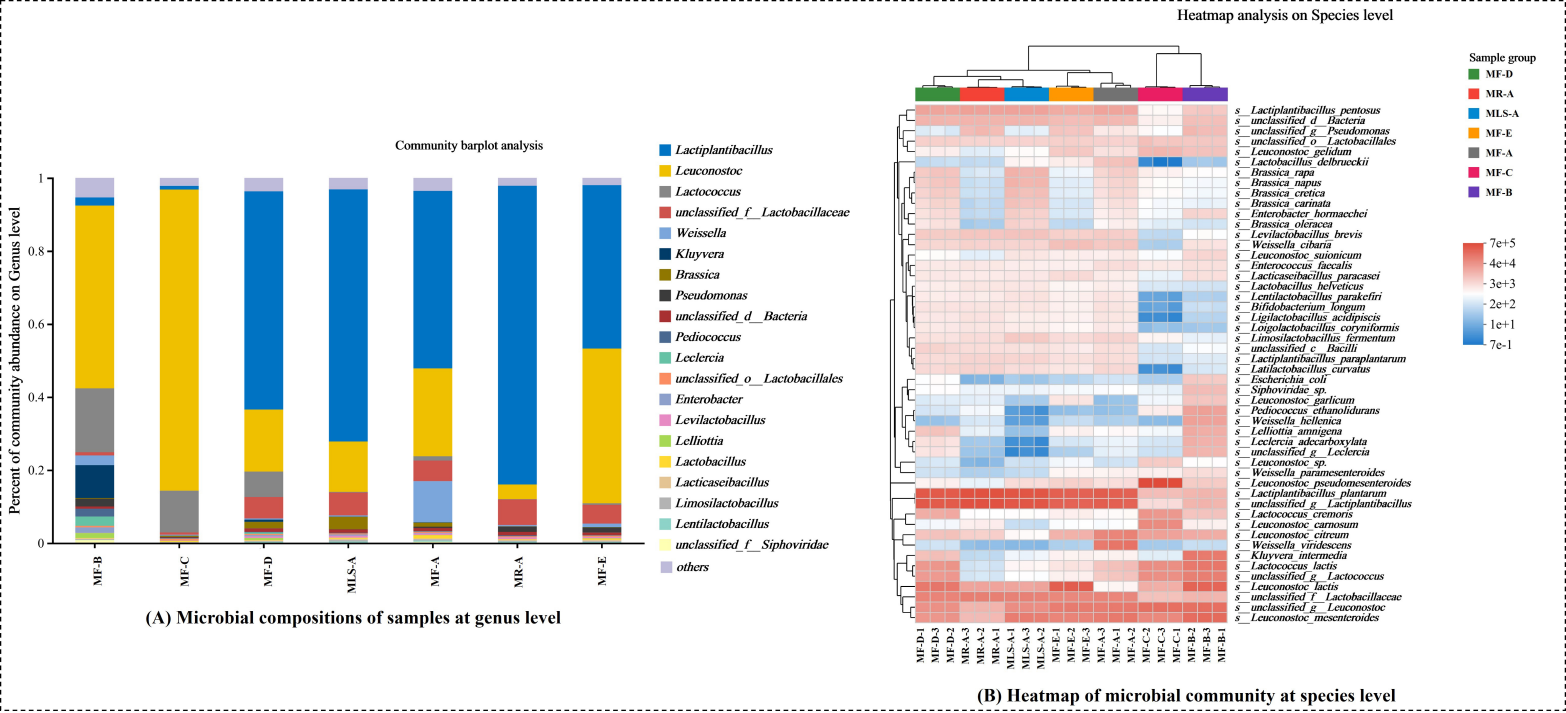
(A) Microbial composition of samples at genus level; (B) Heatmap of the microbial community at the species level. Microbes were determined with three parallels, and the relative abundance of the genus was taken as the average. The top fifty species by relative abundance were shown in the heatmap.

The microbial composition at the genus level is reported in Figure 3A. *Lactiplantibacillus*, *Leuconostoc*, and *Lactococcus* were the dominant microbes across all the samples but with differences in abundance. The microbial compositions differed significantly depending on the substrates/suppliers of ingredients. The relative abundance of *Lactiplantibacillus* was higher in MR-A, which was derived from food and showed good resistance and adhesion in the gastrointestinal tract, and exhibited antioxidant and antimicrobial properties^[40]^. Among the suppliers, *Lactiplantibacillus* was predominant in MF-A, MF-D, and MF-E (44.76%~59.77%), while it was relatively less common in MF-B (2.23%) and MF-C (1.00%). *Lactiplantibacillus* belongs to *Lactobacillus* which is a typical genus of most fermented Suancai and is associated with metabolites of products^[2,3,22]^. The discrepancies in *Lactiplantibacillus* abundance alter the metabolic state and mainly regulate amino acid, lipid, and nucleotide metabolism pathways^[41]^. More importantly, *Lactobacillus* can counteract the combinational coldness and acidity by increasing the biosynthesis of several important biomolecules such as glycolipids and glycoproteins through metabolism^[42]^. It could be inferred that abundant *Lactobacillus* ensures normal fermentation of a product because the coldness and acidity situation is also maintained in non-salt Suancai. *Leuconostoc* is often considered a flavor producer^[43]^ and occupies an important place in MF-C (82.49%). Interestingly, *Weissella* abundance was highest in MF-A, which can drive the diversity and dynamics of the microbial community during fermentation. It helps in the process by its involvement in the transportation and metabolism of amino acids and carbohydrates^[44]^. In addition, *Lactococcus* was involved in the events of low temperature and a series of metabolic reactions through generated proteins^[45]^, which could support the normal fermentation of non-salt Suancai in extreme environments as well, which was highlighted in MF-B, -C, and -D. There was 2.15% *Pseudomonas* in growth in MF-B, which showed a capability to generate 1-octen-3-ol and was associated with ketone production^[46]^.

Furthermore, as shown in Figure 3B, metagenomic analysis could anchor microbial species. *Lactiplantibacillus plantarum*, *Leuconostoc mesenteroides*, *Leuconostoc lactis*, *Leuconostoc citreum*, and *Lactiplantibacillus pentosus* were predominant species in all samples. Among them, *L. plantarum* was the most common species in non-salt Suancai, which is also considered a probiotic to attenuate oxidative stress in neuroblastoma cells^[47]^. Annotations of metabolic pathways suggested that the probiotic *L. plantarum* could alleviate intestinal ischemia-reperfusion damage by modulating calcium-phosphorus homeostasis^[48]^. In addition, the application of *Ln. mesenteroides* increased conjugated linoleic acid and improved the safety^[49]^. In particular, some abundant but unclassified species were identified in the top 50 species, suggesting that there are many unconventional species in non-salt Suancai. Clustering analysis showed that MF-B and MF-C were classified into an identical cluster as a relatively low abundance of *L. plantarum*. Meanwhile, both samples showed lower lactic acid accumulation, which demonstrated that both fermentation mechanisms were different to other samples and related to *L. plantarum* abundance. *Ln. pseudomesenteroides* showed the highest abundance in MF-C, which usually possess the ability to produce exopolysaccharides^[50]^ that might relate to sugar filament appearance.

#### Differences in microbial community structure

LEfSe was a useful tool to distinguish the differential species among samples [linear discriminant analysis (LDA) > 2, *P* < 0.05]. There were 29, 59, and 29 differential species in the samples of different substrates of ingredients. Among them, *L. plantarum* was relatively more common in MR-A, the specific strain of which can reduce total biogenic amines in Dongbei Suancai in Northeast China^[51]^. *Weissella viridescens* possesses thermal resistance^[52]^, which is probably related to its survivability under the boiling process of MF-A. In addition, *Limosilactobacillus fermentum* was highlighted in MLS-A, the specific strain of which is considered a probiotic that can improve intestinal health^[53]^. Among the samples of different suppliers, the exopolysaccharide-producing ability of *Leuconostoc pseudomesenteroides*^[50]^ might contribute to the viscosity of MF-C. Interestingly, different species of *Leuconostoc sp*. were identified in other supplier samples, particularly *Leuconostoc lactis* (MF-E), *Leuconostoc mesenteroides* (MF-B), and *Leuconostoc citreum* (MF-A). These data indicated that the microbial composition varied with the suppliers. It was worth noting that *L. plantarum* was also highlighted in MF-D (LDA = 5.16, *P* < 0.05), which was consistent with cluster analysis [Figure 3B].

**Figure 4:**
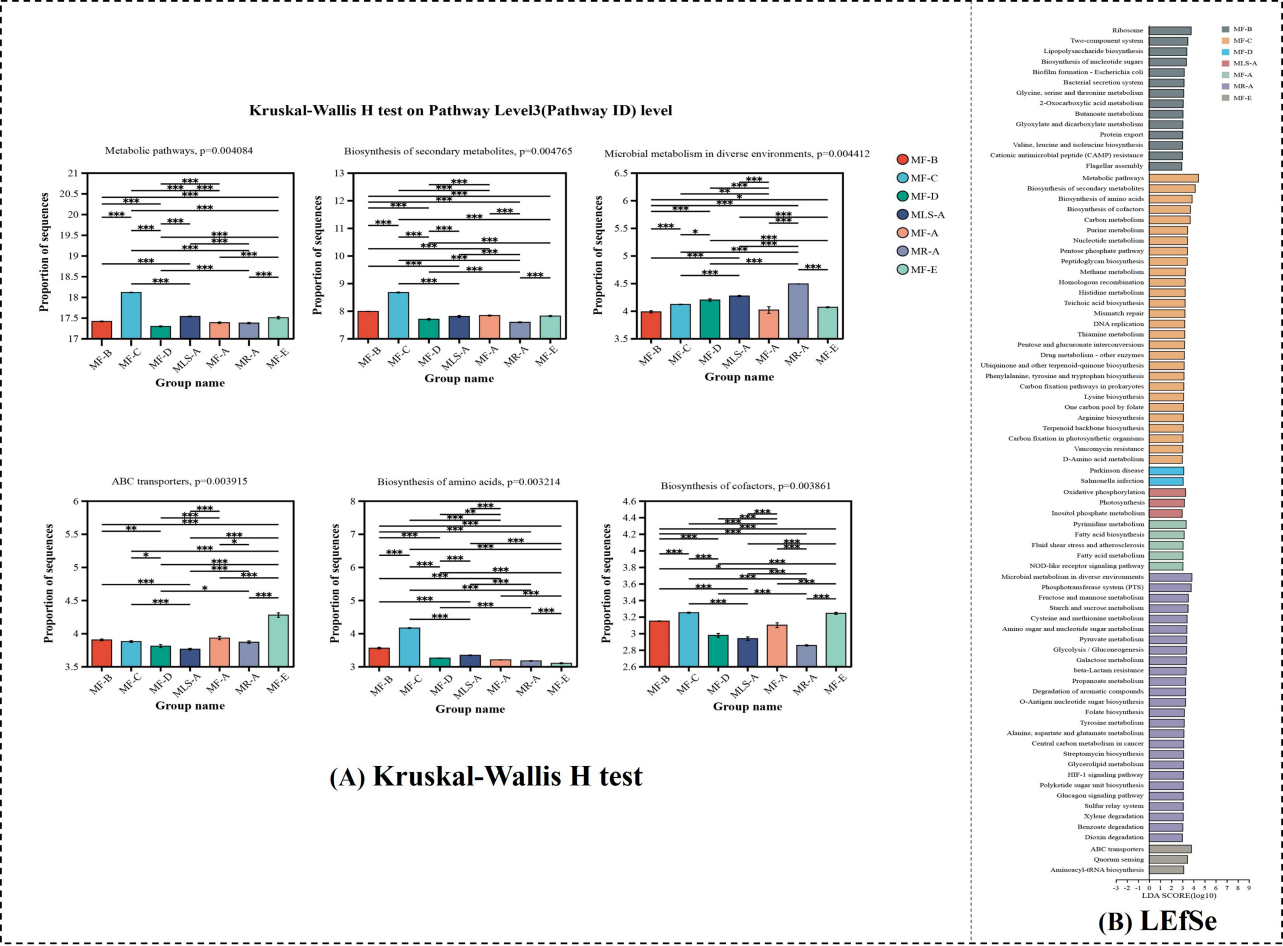
Differential metabolic pathways were calculated by (A) Kruskal-Wallis H test and (B) LEfSe. There were 420 differential pathways whose significance value was less than 0.05, and the abundance of the top six was shown. The differential pathways with LDA > 3,* P* < 0.05 in each sample were shown in different colors. Asterisk refers to statistical significance; ^*^P < 0.05, ^**^P < 0.01, and ^***^P < 0.001. LDA: Linear discriminant analysis.

The Kruskal-Wallis H test was used to reveal the metabolic differences between samples. There were 420 differential pathways with a significance value < 0.05, and the abundance of the top six is shown in Figure 4A. In general, the metabolic pathway was significantly different between samples (*P* < 0.01), which indicated that the metabolic state and metabolic intensity varies with microbial communities. The same goes for the biosynthesis pathway of secondary metabolites (*P* < 0.01), which may affect the diversity of flavor metabolites. The discrepancy of microbial metabolism in different environments (*P* < 0.01) was probably caused by different suppliers, which were similar to the relativity between airborne microorganisms and environmental factors of Baijiu^[54]^. The biosynthesis of amino acids and cofactors contributed to the formation of flavor precursors. In addition, the differential metabolic pathways between samples were calculated based on LEfSe (LDA > 3, *P* < 0.05). As shown in Figure 4B, the pathways of biosynthesis of secondary metabolites, amino acids, cofactors, ubiquinone, and other terpenoid-quinone, *etc.*, were enhanced in MF-C. Thus, the flavor precursors were significantly accumulated. The terpenoid backbone biosynthesis was significantly dominant in MF-C, which contributed to the formation of terpenoid flavor compounds, such as styrene, avocadene, *etc.* The abundant ABC transporters in MF-E contributed to the intracellular metabolism. The starch and sucrose metabolism were active in MR-A, which might provide sufficient substrates for ethanol fermentation^[55]^.

In conclusion, results showed that the total acids and filament’s appearance varied with suppliers that carried different microbiota. Reducing sugars, citric acid, succinic acid, and malic acid were exhausted with fermentation. There were 809 shared metabolites identified by non-target metabolomic analysis, while no exclusive compounds were found between samples. Queuine, cyclopentanol, pc (16:0/0:0), palmitic acid, isorhamnetin, fisetin, and kaempferol 3-o-sophoroside were universally predominant metabolites (the relative content > 3%) in non-salt Suancai. Metagenomic analysis showed that *Lactiplantibacillus plantarum*, *Leuconostoc mesenteroides*, *Leuconostoc lactis*, *Leuconostoc citreum*, and *Lactiplantibacillus pentosus* were the most predominant species in all samples. There were 420 differential pathways with a significance value < 0.05 based on the Kruskal-Wallis H test. Taken as a whole, the microbial community and its metabolism of non-salt Suancai varied with the substrates/suppliers of ingredients. This information is practically important for the inheritance of traditional technology and quality control.

## DECLARATIONS

### Copyright

© The Author(s) 2024.
