# Supplementary material for: Effects of substrates and suppliers of ingredients on microbial community and metabolites of traditional non-salt Suancai
Source: Microbiome Res Rep. 2024 Mar 13;3(2):21. doi: 10.20517/mrr.2023.76 (PMC11149085; doi:10.20517/mrr.2023.76)
Supplement: Supplementary file 1 [file mrr-3-2-21-SupplementaryMaterials.pdf]

Lai *et al. Microbiome Res Rep* 2024;3:21
**DOI:** 10.20517/mrr.2023.76

**Microbiome Research Reports**

# Supplementary Materials

## Effects of substrates and suppliers of ingredients on microbial community and metabolites of traditional non-salt Suancai

**Haimei Lai[1], Lang Yan[2], Yali Wang[1], Yuan Mei[1], Yuli Huang[1], Xueqing Zeng[1], Lihong Ge[3], Jichun Zhao[4], Yongqing Zhu[1], Qiaolian Huang[1], Menglu Yang[1], Nan Zhao[1]**

[1]Institute of Agro-products Processing Science and Technology, Sichuan Academy of Agricultural Sciences, Chengdu 610066, Sichuan, China.
[2]Panxi Crops Research and Utilization Key Laboratory of Sichuan Province, Xichang College, Xichang 615000, Sichuan, China.
[3]College of Life Science, Sichuan Normal University, Chengdu 610066, Sichuan, China.
[4]College of Food Science, Southwest University, Chongqing 400715, China.

**Correspondence to:** Dr. Menglu Yang, Dr. Nan Zhao, Institute of Agro-products Processing Science and Technology, Sichuan Academy of Agricultural Sciences, No. 60, Shizishan Road, Jinjiang District, Chengdu 610066, Sichuan, China. E-mail: MengluYY@126.com; demonzn1115@163.com

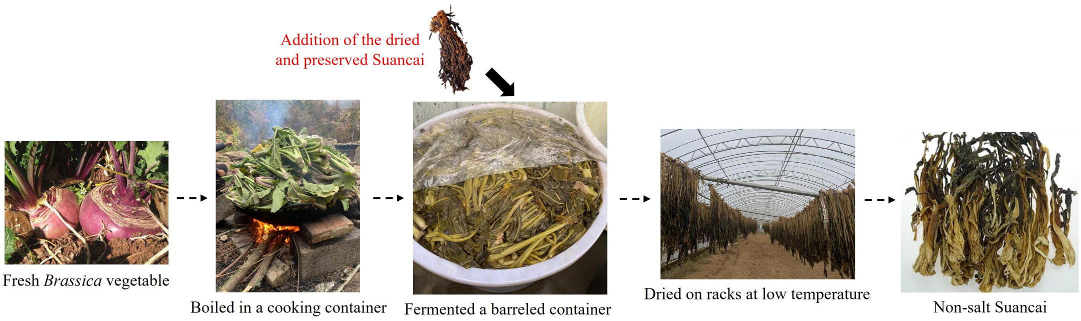


**Supplementary Figure 1.** A diagram of traditional technology of non-salt Suancai.

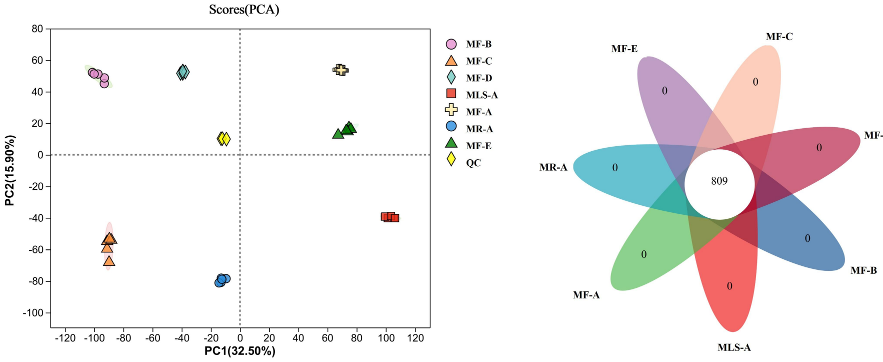


(A) PCA diagrams among experimental and QC samples

(B) Venn plot of identified metabolites

**Supplementary Figure 2.** (A) PCA plot of samples; (B) Venn diagram of identified metabolites

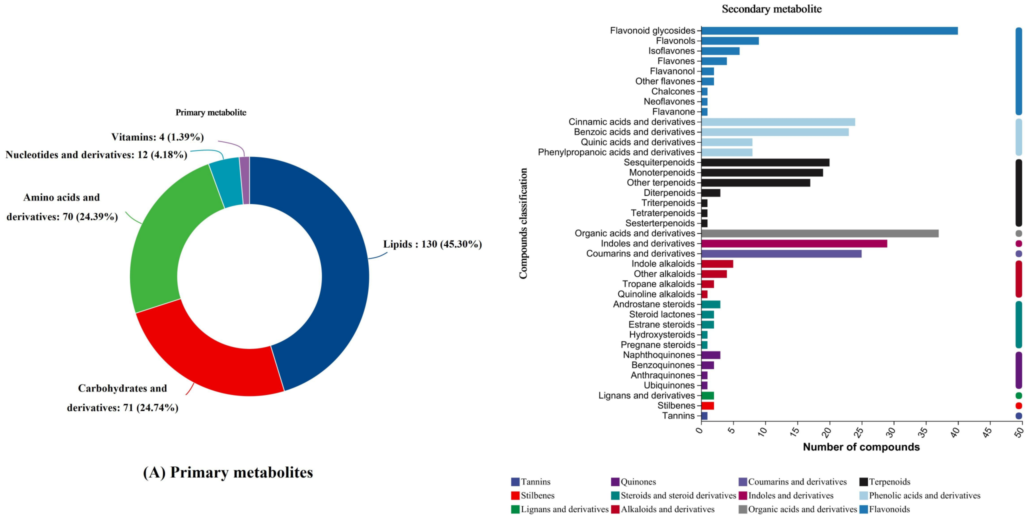


(A) Primary metabolites

(B) Secondary metabolites

**Supplementary Figure 3.** Metabolites were classified into (A) primary and (B) secondary.

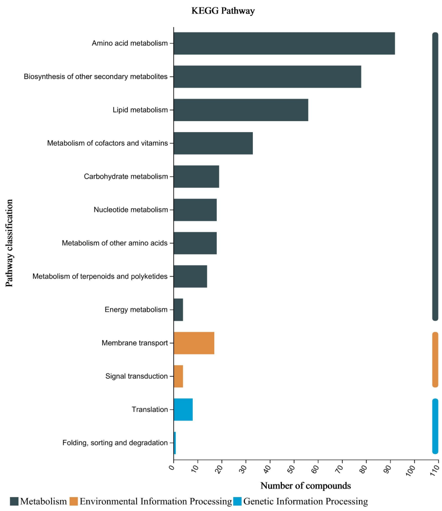


(A) KEGG pathway

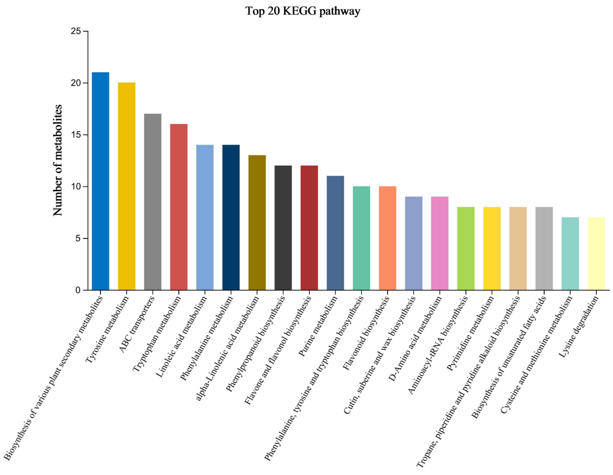


(B) Important pathway aggregation based on KEGG database (top 20)

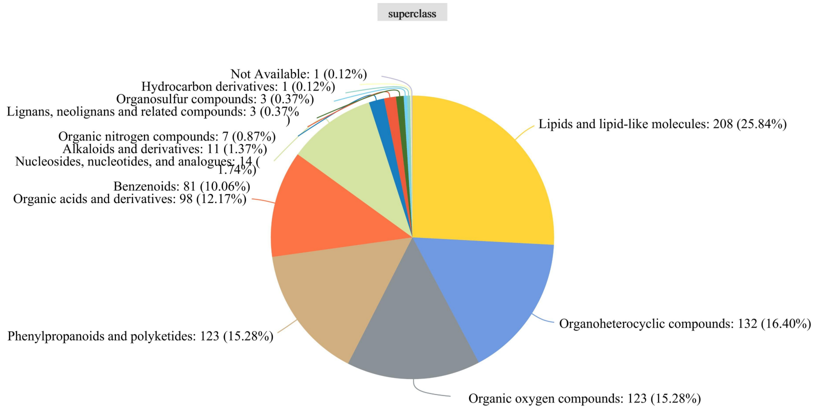


(C) Pathways matched to HMDS database

**Supplementary Figure 4.** Metabolites were enriched through the metabolic pathway of (A) KEGG database and its (B) important pathway, (C) HMDS database.

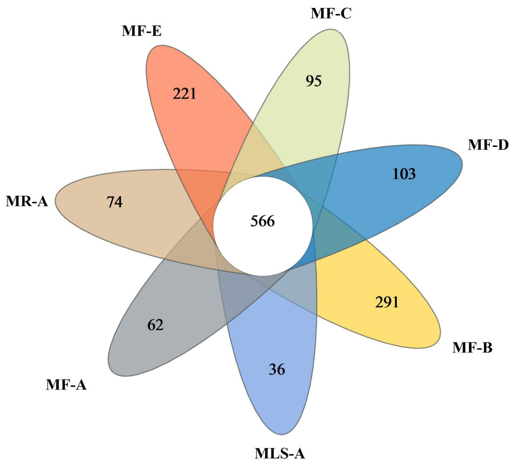


Venn diagram of microbes at species level

**Supplementary Figure 5.** Veen diagram of microbes among samples at species level.

**Supplementary Table 1. Mobile phase elution gradient of C18 column**

| **Time (min)** | 0 | 0.5 | 7.5 | 13 | 14.4 | 14.5 | 16 |
|---|---|---|---|---|---|---|---|
| **A%** | 98 | 98 | 65 | 5 | 5 | 98 | 98 |
| **B%** | 2 | 2 | 35 | 95 | 95 | 2 | 2 |

**Supplementary Table 2. Raw and annotated data for metabolomics**

| **Ion mode** | **All peaks** | **Identified metabolites** | **Metabolites in library** | **Metabolites in KEGG** |
|---|---|---|---|---|
| Pos | 8779 | 510 | 506 | 312 |
| neg | 7907 | 317 | 313 | 197 |
| mix | 16686 | 827 | 819 | 509 |

All peaks mean the number of MS peak selected by software. Identified metabolites mean the final number of metabolites identified by self-built, Metlin, HMDB database, *etc*. Metabolites in Library means the number of metabolites blast by using the common database, such as HMDB, Lipidmaps, *etc*. Metabolites in KEGG means the number of metabolites blast by using the KEGG database.

**Supplementary Table 3. Raw and annotated data for metagenomics**

| Samples | Raw reads | Raw base (bp) | Clean reads | Clean base (bp) | Contigs | ORFs |
|---|---|---|---|---|---|---|
| MF-B-1 | 46,565,648 | 7,031,412,848 | 46,189,400 | 6,963,961,674 | 61,092 | 104,769 |
| MF-B-2 | 46,007,430 | 6,947,121,930 | 45,696,036 | 6,888,026,046 | 65,427 | 110,566 |
| MF-B-3 | 50,701,468 | 7,655,921,668 | 50,345,484 | 7,588,961,304 | 63,647 | 108,753 |
| MF-C-1 | 40,780,878 | 6,157,912,578 | 40,455,630 | 6,101,538,479 | 13,989 | 25,981 |
| MF-C-2 | 43,598,670 | 6,583,399,170 | 43,289,718 | 6,528,807,247 | 17,022 | 28,888 |
| MF-C-3 | 439,243,56 | 6,632,577,756 | 43,614,908 | 6,578,313,515 | 15,063 | 27,102 |
| MF-D-1 | 44,531,978 | 6,724,328,678 | 44,204,116 | 6,666,575,009 | 51,456 | 69,416 |
| MF-D-2 | 51,208,942 | 7,732,550,242 | 50,914,276 | 7,676,236,289 | 72,635 | 82,160 |
| MF-D-3 | 46,279,170 | 6,988,154,670 | 45,919,582 | 6,923,416,810 | 64,432 | 80,234 |
| MR-A-1 | 44,499,482 | 6,719,421,782 | 44,162,520 | 6,656,337,359 | 82,205 | 65,725 |
| MR-A-2 | 45,552,980 | 6,878,499,980 | 45,196,236 | 6,814,750,726 | 66,550 | 56,929 |
| MR-A-3 | 44,245,956 | 6,681,139,356 | 43,909,272 | 6,618,484,396 | 65,129 | 53,886 |
| MF-A-1 | 42,258,222 | 6,380,991,522 | 41,940,690 | 6,323,216,959 | 33,205 | 41,747 |
| MF-A-2 | 41,446,412 | 6,258,408,212 | 41,119,276 | 6,198,068,708 | 35,522 | 42,715 |
| MF-A-3 | 42,396,386 | 6,401,854,286 | 42,082,210 | 6,343,650,402 | 24,233 | 34,652 |
| MLS-A-1 | 43,028,758 | 6,497,342,458 | 42,744,956 | 6,447,383,956 | 13,986 | 28,968 |
| MLS-A-2 | 47,400,278 | 7,157,441,978 | 47,083,872 | 7,100,564,733 | 16,962 | 33,031 |
| MLS-A-3 | 41,977,944 | 6,338,669,544 | 41,695,654 | 6,287,949,718 | 13,850 | 28,805 |
| MF-E-1 | 43,584,022 | 6,581,187,322 | 43,273,986 | 6,526,131,944 | 26,546 | 40,508 |
| MF-E-2 | 46,761,246 | 7,060,948,146 | 46,407,778 | 6,997,117,946 | 27,413 | 41,747 |
| MF-E-3 | 41,402,248 | 6,251,739,448 | 41,177,482 | 6,207,496,294 | 27,440 | 41,023 |

**Supplementary Table 4. Chao 1 and Simpson index of the determined samples**

| Sample | Chao 1 | Simpson |
|---|---|---|
| MF-A-1 | 1618 | 0.152 |
| MF-A-2 | 1606 | 0.142 |
| MF-A-3 | 1548 | 0.170 |
| MLS-A-1 | 898 | 0.258 |
| MLS-A-2 | 873 | 0.268 |
| MLS-A-3 | 890 | 0.266 |
| MR-A-1 | 1237 | 0.351 |
| MR-A-2 | 1315 | 0.349 |
| MR-A-3 | 1210 | 0.352 |
| MF-B-1 | 2256 | 0.112 |
| MF-B-2 | 2310 | 0.109 |
| MF-B-3 | 2312 | 0.112 |
| MF-C-1 | 1383 | 0.376 |
| MF-C-2 | 1494 | 0.365 |
| MF-C-3 | 1438 | 0.376 |
| MF-D-1 | 1654 | 0.216 |
| MF-D-2 | 1751 | 0.199 |
| MF-D-3 | 1774 | 0.200 |
| MF-E-1 | 1559 | 0.198 |
| MF-E-2 | 1632 | 0.197 |
| MF-E-3 | 1610 | 0.198 |
